# Management of High Urogenital Sinus in Adults: A Scoping Review

**DOI:** 10.3390/medicina61020191

**Published:** 2025-01-22

**Authors:** Muhammed A. Moukhtar Hammad, Nardeen Magdy Samaan, Gamal Ghoniem

**Affiliations:** 1 Department of Urology, University of California Irvine, Orange, CA 92868, USA; mahammad@hs.uci.edu; 2 Nasser Institute for Research and Treatment, Cairo 4350210, Egypt; nardeenaziz@gmail.com

**Keywords:** persistent urogenital sinus, high urogenital sinus, feminizing genitoplasty, congenital adrenal hyperplasia, vaginal reconstruction

## Abstract

*Background and Objectives:* Persistent urogenital sinus (PUGS) is a rare congenital anomaly resulting from disruptions in urogenital development during embryogenesis. Characterized by the confluence of the vaginal canal and urethra into a single channel, it poses diagnostic and management challenges, particularly in adult patients. Surgical correction remains the primary treatment at an early age, yet there are no universally accepted guidelines regarding treatment at later stages in life. We explored current evidence on the management of high urogenital sinus (HUGS) in adults, focusing on surgical techniques, outcomes, perioperative care, and follow-up. *Materials and Methods:* A scoping review was conducted using PubMed, Scopus, and Google Scholar, including studies published up to August 2024. Search terms included “high urogenital sinus”, “adult patients”, and related conditions. Articles were included if they addressed clinical, diagnostic, or therapeutic data on HUGS in adult populations. Data extraction was performed independently by two reviewers, and a PRISMA-ScR flow diagram was used to outline the study selection process. *Results:* Nine studies met inclusion criteria, with data on surgical techniques, perioperative care, and long-term outcomes extracted for analysis. The included studies highlighted diverse surgical approaches, such as feminizing genitoplasty, buccal mucosa vaginoplasty, posterior prone techniques, and robotic-assisted urogenital sinus mobilization. Complications like vaginal stenosis, urethrovaginal fistula, and urinary incontinence were reported. Long-term outcomes were under-reported, with limited data on sexual function and patient satisfaction. Hormonal therapies, including glucocorticoids, played critical roles in managing cases associated with congenital adrenal hyperplasia (CAH). *Conclusions:* The management of HUGS requires a multidisciplinary approach integrating advanced surgical techniques and individualized perioperative care. While short-term outcomes are generally favorable, long-term data on sexual function and quality of life remain scarce. Future research should prioritize longitudinal studies to address these gaps, aiming to optimize surgical outcomes, patient satisfaction, and psychosocial well-being.

## 1. Introduction

Occurring at an incidence of approximately 6 in 100,000 women, urogenital sinus (UGS) malformation, also known as persistent urogenital sinus (PUGS), is a rare congenital pathology [1,2]. It occurs when the vaginal canal merges with the urethra into a single channel rather than opening into a distinct vulva. It generally arises from disruptions in urogenital development during crucial stages of embryogenesis. The confluence can occur either near the bladder neck (high UGS or HUGS) or closer to the external opening (low UGS). Currently, there are no universally accepted guidelines for managing UGS, including the timing of surgery and long-term patient follow-up.

### Embryology

With the cloacal membrane rupturing to create the urogenital plate on the surface of the perineum, the cloaca divides to form the urogenital sinus [3]. At the 12th–16th weeks of gestation, the junction between the developing lower vagina and urogenital sinus gradually shifts caudally, eventually reaching the posterior wall of the urogenital sinus and detaching from the urethra in the vestibule to form a separate vaginal opening [4].

While some studies suggest that cases of PUGS may be due to renal duct hypoplasia or inadequate growth of the tail urogenital wedge, the majority attribute the cases to the high androgen levels stimulated by congenital adrenal hyperplasia (CAH) and the location of the vaginal confluence to the descending position of the sinus ridge, which is related to androgen levels [5,6,7,8,9]. Prenatal exposure to androgens in female mice has been found to inhibit the descent of the sinus ridge and prevent the formation of the vaginal opening in the vulva [10]. Additionally, recent findings indicate that the extent of androgen exposure impacts the position of the sinus ridge, with higher androgen levels causing a proximal shift in the vaginal and urethral junction, closer to the bladder neck in rodents [11,12,13].

It is likely that PUGS is caused by a combination of genetic, hormonal, environmental, and mechanical factors. The specific factors involved may vary depending on the individual case. Due to the complexity of normal urogenital differentiation, adult cases of high UGS are infrequent. Surgery remains the primary treatment and is typically carried out in childhood.

However, there are still significant gaps in understanding the causes, ideal management, and long-term outcomes of HUGS in adults. We therefore aimed to explore the available literature for related articles on the management of HUGS in adult women.

## 2. Methodology

### 2.1. Search Strategy and Study Selection

A scoping review of the literature was conducted to identify relevant studies on HUGS in adults. The search was performed using PubMed, Scopus, and Google Scholar, covering studies published up to August 2024. The search terms included variations of “high urogenital sinus”, “adult patients”, and other related urological conditions. Boolean operators (AND, OR) were used to combine key terms, and the search was limited to articles in English and adult human subjects.

Two independent reviewers performed the literature search and screened the titles and abstracts of all identified articles. Studies were included if they met the following criteria: (1) focused on HUGS in female adult populations; (2) provided clinical, diagnostic, or therapeutic data; and (3) included original research, case series, cohort studies, reviews, or editorials. Articles were excluded if they involved pediatric populations exclusively or were non-English. Discrepancies between reviewers were resolved through discussion or consultation with a third reviewer.

### 2.2. Data Extraction and Quality Assessment

The two reviewers independently extracted data from the selected studies using a standardized form. The extracted data included study characteristics (authors, year, study design, sample size), patient demographics, clinical presentations, diagnostic modalities, and outcomes related to the treatment and management of high urogenital sinus.

### 2.3. PRISMA Flow Diagram

**Figure 1:**
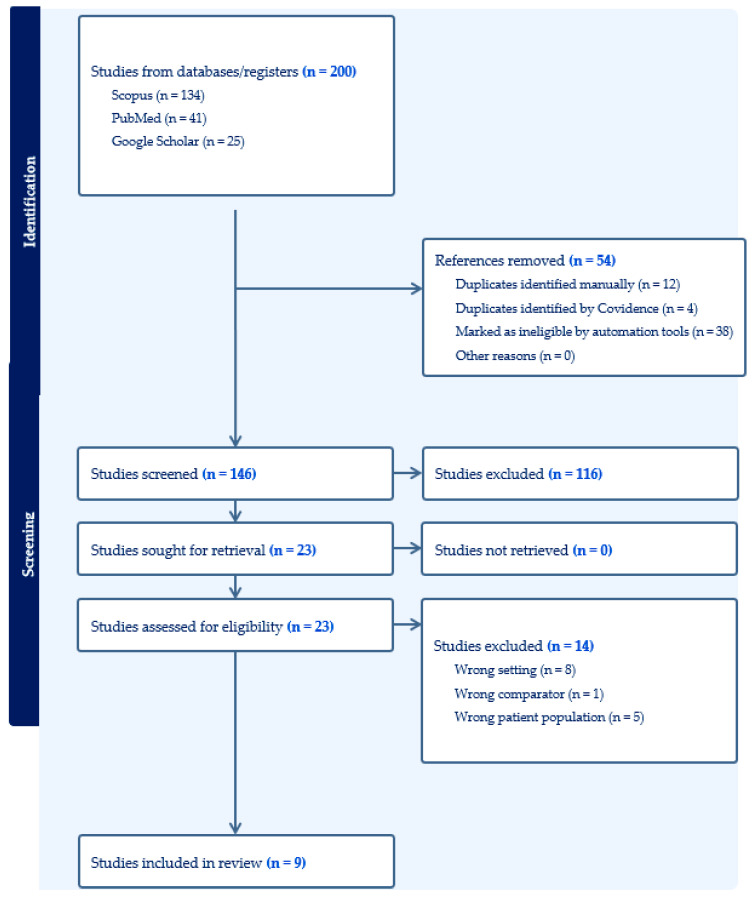
PRISMA-ScR (Preferred Reporting Items for Systematic reviews and Meta-Analyses extension for Scoping Reviews) flow chart.

The PRISMA-ScR (Preferred Reporting Items for Systematic reviews and Meta-Analyses extension for Scoping Reviews) flow diagram illustrates the study selection process (Figure 1). Initially, 200 articles were retrieved through database searches. After the removal of duplicates, 146 articles were screened by title and abstract, with 116 being excluded due to irrelevant titles or failure to meet inclusion criteria. Finally, 23 studies were assessed for eligibility, of which 9 were included in the final analysis after excluding articles with an unrelated study population or wrong setting.

## 3. Results

Nine studies met our inclusion and exclusion criteria (Table 1). With limited literature on PUGS surgical data, most of the available data are retrospective and based on a small number of cases, focusing mainly on short-term outcomes such as the appearance of the vulva and postoperative complications (Table 2).

In reviewing the management of urogenital sinus anomalies across various studies, a range of approaches and outcomes were presented. Braz (1999) highlighted the use of vaginal replacement with a sigmoid colon in eight patients, noting a complication of urethrovaginal fistula in one case, which was successfully corrected [14]. This was followed by a report from Mindy L. Samuelson et al. (2006) on the management of a single patient with adrenogenital syndrome, where feminizing genitoplasty and buccal mucosa vulvovaginoplasty were performed without complications [15].

Podesta et al. (2008) contributed by examining 12 cases involving urogenital sinus anomalies in disorders of sex development (DSD) patients. While the surgical outcomes were generally favorable, some patients experienced glans atrophy, vaginal stricture, and urinary incontinence [16]. These findings were contrasted by Bailez et al. (2014), who described 55 cases of CAH treated with a urogenital sinus mobilization maneuver, reporting no adverse outcomes and no impact on voiding function [17].

Sircili et al. (2016) focused on 20 patients with CAH who had undergone failed previous surgeries [18]. A Y-V perineal flap technique was employed, and although one patient required reoperation, most patients had favorable outcomes, including reports of pain-free sexual activity.

In a more recent study by Wang et al. (2021), a patient with persistent urogenital sinus and associated anatomical anomalies was treated with urethral reconstruction and artificial vaginoplasty, resulting in successful urination and long-term management without complications [19]. Around the same time, Oktay Ulusoy et al. (2021) explored a posterior prone approach for HUGS, confirming normal pelvic function postoperatively, supported by electromyography [20].

Ellerkamp et al. (2021) offered further insights into the treatment of secondary vaginal stenosis after prior surgeries, achieving positive outcomes through partial urogenital mobilization [21]. Yang et al. (2023), on the other hand, described the use of robotic techniques in a complex case involving VACTERL syndrome, achieving successful outcomes without complications [22]. Each of these studies contributes to an evolving understanding of the best approaches for managing HUGS, offering a wide array of techniques with generally positive results.

## 4. Discussion

The management of HUGS represents a challenge, particularly in adult patients, where the condition often presents with complications from delayed diagnosis or failed pediatric interventions. Its surgical management often includes a variety of surgical approaches tailored to the anatomical complexities and associated conditions of each patient. Different management strategies and techniques are described in the literature.

### 4.1. Feminizing Genitoplasty and Buccal Mucosa Vaginoplasty

Samuelson et al. (2005) reported a case where a patient with HUGS and adrenogenital syndrome underwent feminizing genitoplasty combined with buccal mucosa vulvovaginoplasty [15]. This approach aimed to reconstruct the external genitalia to a typical feminine appearance while addressing functional aspects. Buccal mucosa grafts were utilized due to their favorable healing properties and resistance to stenosis. The combination enhanced both functional and aesthetic outcomes.

### 4.2. Feminizing Genital Reconstruction with Long-Term Follow-Up

Podesta et al. (2008) treated 12 patients with HUGS and DSD using feminizing genital reconstruction [16]. Preoperative evaluations included history taking, clinical examination, laboratory tests, karyotyping, ultrasound, and radiological examinations with urethroscopy. Postoperative complications such as glans atrophy, vaginal stricture, and urinary stress incontinence were noted. Despite these, a successful feminine genital appearance was achieved, emphasizing the need for long-term follow-up to monitor and manage late complications.

### 4.3. Perineal Prone Approach Without Division of the Rectum

Rink et al. (1997) described a perineal prone approach for repairing HUGS without dividing the rectum [23]. This technique was employed in eight cases and allowed for good access to the urogenital tract while minimizing trauma to surrounding structures. Some patients experienced urethrovaginal fistulas and mild vaginal stenosis, which were managed successfully with dilation. Although it has been extensively studied with consistent long-term outcomes, it involves more invasive steps, such as rectal division, and results in longer recovery periods and higher complication rates, including urinary or fecal incontinence in some cases.

### 4.4. Partial Urogenital Mobilization and Flap Vaginoplasty

Ellerkamp et al. (2015–2018) treated 13 patients with DSD who developed vaginal stenosis after previous surgeries [21]. Management involved partial urogenital mobilization with perineal or lateral flaps and, in some cases, bowel vaginoplasty. One patient required a revision vaginoplasty, which failed due to complex anatomy. The study emphasized detailed anatomical assessment and appropriate surgical techniques to minimize complications.

### 4.5. Posterior Prone Approach with Pelvic Muscle Sparing

Ulusoy et al. (2021) managed seven patients with HUGS using a posterior prone approach without dividing the rectum, focusing on preserving pelvic floor muscle function [20]. Postoperative assessments using EMG–uroflowmetry showed normal pelvic floor function, indicating that this technique effectively preserves urinary function.

### 4.6. UGS Mobilization Maneuver

Bailez et al. (2014) reported on 55 cases of patients with CAH, treated with the UGS mobilization maneuver [17]. In patients with HUGS, a combination of pull-through techniques with balloon catheter placement (e.g., Foley catheter) and principles of the anterior sagittal transrectal approach (ASTRA) method was used. This technique did not compromise voiding function or urinary continence.

### 4.7. Robotic Total Urogenital Sinus Mobilization

Yang et al. (2023) described a novel approach using robotic total urogenital sinus mobilization combined with posterior sagittal anorectoplasty in a patient with VACTERL syndrome and a bicornuate uterus [22]. Robotic surgery allowed for the precise dissection and mobilization of urogenital structures, reducing intraoperative trauma and enhancing visibility in complex anatomical fields. Early results demonstrated promising outcomes without significant complications. A higher technical demand and dependency on surgeon expertise with robotics may influence variability in outcomes, especially in centers with limited experience

### 4.8. Vaginal Reconstruction and Long-Term Dilation

Bailez et al. (1992) conducted staged vaginal reconstruction with salt-wasting adrenal hyperplasia [24]. The initial procedure involved opening the urogenital sinus and exteriorization of the vagina through a perineal approach. A significant proportion (78.5%) required further vaginal reconstructive procedures to achieve a normal vaginal outlet. The authors highlighted the necessity of long-term follow-up and periodic dilation to prevent vaginal stenosis. Moreover, Ghoniem et al. (2024) reported a 21-year-old female misdiagnosed with transverse vaginal septum initially [25]. Her medical history included bilateral hydroureteronephrosis, leading to surgical interventions such as ureteric reimplantation and bladder flap surgery. Upon examination, her genitalia showed an HUGS, prompting further investigation. A modified ASTRA technique was used to surgically correct it, with steps taken to identify and close a fistula and lengthen the vaginal canal using skin flaps. Leuprolide acetate was administered to suppress menstruation and prevent complications. Postoperatively, the patient had no infections or incontinence but required serial vaginal dilations to prevent stenosis, achieving success with no further interventions after 12 months.

Robotic modalities typically involve higher initial costs due to expensive equipment, maintenance, and training requirements. However, shorter hospital stays and reduced postoperative complications might offset these costs over time. Other approaches are generally less expensive upfront but may lead to higher long-term costs if complications or revisions are required. For instance, cases requiring multiple follow-ups for vaginal stenosis or fistula repair could significantly increase the total cost, but limited cost-effectiveness data comparing these modalities exist. Future studies should evaluate both direct (e.g., surgical expenses) and indirect costs (e.g., recovery time, quality of life impacts).

### 4.9. Perioperative Care: Hormonal Treatment

Since most PUGS cases are secondary to CAH, their main treatment besides surgical female genital reconstruction is long-term glucocorticoid therapy, suppressing excess hormones, replacing deficient hormones, and avoiding plausible Cushing-like side effects [26]. Estrogen replacement therapy with progesterone should be commenced around physiological puberty to induce periodic bleeding (menstruation) and gradual transition to adulthood [27].

For patients with a normal hormonal profile, it is recommended to receive gonadotropin-releasing hormone (GnRH) agonists for menstrual suppression perioperatively. GnRH agonists are non-contraceptive medications used for menstrual suppression by creating a hypoestrogenic state. Depot leuprolide acetate 11.25 mg, injected intramuscularly every 12 weeks, is commonly used [28,29]. Rates of amenorrhea are as high as 96% [28,30]. Patients should be counseled to expect an initial “flare” in bleeding within 1 to 3 weeks until hormone levels are adequately suppressed [30]. Add-back therapy (e.g., norethindrone acetate 5 mg once daily) can help to minimize other bothersome symptoms [28].

### 4.10. Follow-Up Outcomes

Ellerkamp et al. reported that perineal flaps with partial urogenital mobilization provided normal anatomical results with normal sexual function in patients following female genital repair [21]. In contrast, other studies reported unsatisfactory follow-up outcomes after female genital reconstruction. A meta-analysis reported impaired clitoral sensitivity, vaginal stenosis, and pain and discomfort during intercourse [31]. Two other studies with long-term follow-up showed that postoperative outcomes with CAH in terms of sexual function and clitoral sensitivity were unsatisfactory [32,33].

A major etiology that complicates the outcome and success of vaginal reconstruction is patient participation with vaginal dilation. Stenosis is common after vaginal reconstruction, and long-term postoperative dilation is needed to prevent it. The ability to self-dilate the vagina or neovagina requires an understanding of one’s own anatomy, the reason for the procedure, and the necessity for long-term dilation. In the early teens, this is physically, emotionally, and psychologically challenging, often resulting in non-compliance and subsequent vaginal stenosis. A thorough interdisciplinary approach with gynecology, surgery, urology, and psychology teams on board is essential for assessing the patient’s readiness and ability to comply with dilation protocols [34].

Although patient data are heterogeneous regarding diagnosis, age, and surgical method, the success of vaginoplasty outcomes is improved when performed after puberty [35,36,37]. This improvement is believed to stem from better compliance with dilation and the estrogenization of tissue during the postoperative healing process. Many recommend undertaking these procedures when patients are ready to initiate vaginal sexual activity [35,36,38].

Long-term outcomes are, however, under-reported in the literature. Developing dedicated registries could be of particular benefit.

### 4.11. Psychological Well-Being and Support System

Determining readiness for complex surgical reconstruction during pubertal maturation requires assessment of the patient’s psychological well-being and available support. Although individuals with complex anatomies such as cloacal exstrophy and cloacal malformation have other comorbidities, they are reported to have good psychological functioning [39]. However, adolescents may have anxiety about their genital appearance and sexual activity [40]. A close follow-up and endorsing family support may contribute to a successful outcome. These complex cases should also be referred to experienced pelvic floor reconstruction surgeons. The utilization of different techniques in the same patient is frequent and familiarity of these techniques optimizes the outcome. This interdisciplinary approach is a crucial component of perioperative care.

The management of HUGS encompasses various surgical approaches, each presenting unique strengths and challenges. Advances such as the posterior prone approach and robotic urogenital sinus mobilization reflect a broader trend toward minimally invasive surgery, which aims to enhance visualization and minimize tissue trauma [22,23]. From a clinical perspective, the selection of surgical technique should prioritize not only anatomical correction but also functional outcomes, including urinary continence, sexual function, and quality of life.

The optimal approach must consider patient-specific factors, such as the severity of anatomical anomalies and prior interventions. Robotic surgery offers precision and shorter recovery times but requires significant resources and surgeon expertise, limiting its availability in resource-constrained settings. In contrast, traditional perineal approaches remain effective but may involve longer recovery and higher risks of complications, such as urinary or fecal incontinence [20]. Despite advancements in surgical techniques, long-term outcomes remain under-reported in the literature. While many studies focus on immediate postoperative results, there are limited data on the durability of surgical corrections, sexual function, and patient satisfaction over time. Future studies should systematically compare these metrics to provide a standard guidance on their relative benefits in different clinical scenarios.

## 5. Limitations

Some studies include fewer than 10 patients, while others analyze larger cohorts (e.g., 55 cases in Bailez et al. [17]). Techniques vary widely, ranging from single-stage approaches using posterior prone mobilization to more complex multi-stage procedures with bowel vaginoplasty. This variability makes a direct comparison of outcomes challenging. A standardized approach to data collection and consistent classification systems (e.g., Prader scores or UGS length thresholds) would improve comparative analyses. Longitudinal studies with uniform endpoints, such as continence, cosmesis, and sexual functionality, are needed.

## 6. Conclusions

The management of PUGS remains complex due to its rare occurrence and the diversity of anatomical presentations. Advances in diagnostic imaging and surgical techniques have significantly improved our ability to assess and treat this congenital condition. Despite these developments, long-term outcomes, particularly concerning sexual function and patient satisfaction, remain underexplored, and further high-quality, longitudinal studies are required. While hormonal therapy and perioperative care play essential roles in the overall treatment of PUGS, given the complexity of these cases, a multidisciplinary approach involving urology, gynecology, endocrinology, and psychology is critical for optimizing outcomes. Future research should aim to improve long-term data collection, focusing on sexual function, quality of life, and the psychosocial well-being of patients undergoing these life-altering procedures, which could be implemented via multicenter studies and registries of this relatively rare condition.

## Figures and Tables

**Table 1:** General characteristics and methods summary of the studies included.

No.	Author(s)	Year	Diagnosis	Method of Diagnosis	Management
1	Braz, A [14]	1999	Urogenital sinus, high vaginal implantation, normal rectum, sigmoidostomy	Cystoscopic and radiological examinations	Vaginal replacement with sigmoid colon, protective sigmoidostomy
2	Samuelson et al. [15]	2006	Mid-to-high urogenital sinus in adrenogenital syndrome	Clinical examination, endoscopic evaluation	Feminizing genitoplasty, buccal mucosa vulvovaginoplasty
3	Podesta, M et al. [16]	2008	Urogenital sinus anomalies in DSD	Clinical history, examination, laboratory tests, karyotyping, ultrasound, radiology, urethroscopy	Feminizing genital reconstruction
4	Bailez, M.M. et al. [17]	2014	Congenital adrenal hyperplasia (CAH) with intermediate and high urogenital sinus	Contrast imaging studies	Urogenital sinus mobilization maneuver
5	Sircili, M.H.P. et al. [18]	2016	Congenital adrenal hyperplasia (CAH) with failed previous surgery	Cystoscopy	Y-V perineal flap with/without partial mobilization of urogenital sinus
6	Wang et al. [19]	2021	Persistent urogenital sinus (PUG) with uterus didelphys and double vagina	Transabdominal ultrasound, transrectal ultrasound, contrast-enhanced ultrasound	Urethral reconstruction, vaginal pull-through, artificial vaginoplasty, bilateral hysterosalpingectomy
7	Ulusoy et al. [20]	2021	High urogenital sinus	Clinical and imaging evaluation	Posterior prone approach for repair without rectal division
8	Ellerkamp, V et al. [21]	2021	Secondary vaginal stenosis after reconstructive surgery for urogenital sinus anomalies	Genitoscopy, genitography, MRI	Perineal flap with partial urogenital mobilization
9	Yang et al. [22]	2023	VACTERL syndrome, urinary tract infection, high urogenital sinus, bicornuate uterus	Ultrasound, voiding cystourethrogram (VCUG), MRI	Vaginoplasty, robotic urogenital sinus mobilization, posterior sagittal anorectoplasty

**Table 2:** Complications and clinical outcomes.

No.	Author(s)	Complications	Number of Cases	Remarks/Outcome
1	Braz, A [14]	Urethrovaginal fistula (re-operated and cured)	8	One patient successfully engaged in sexual intercourse post-vaginal-replacement.
2	Samuelson et al. [15]	None reported	1	-
3	Podesta, M et al. [16]	Glans atrophy, vaginal stricture, urinary stress incontinence	12	Achieved a satisfactory feminine genital appearance; long-term follow-up needed.
4	Bailez, M.M. et al. [17]	None reported	55	Does not compromise voiding function or urinary continence.
5	Sircili, M.H.P. et al. [18]	One patient required a second reoperation	20	Eight adult patients were sexually active without experiencing dyspareunia.
6	Wang et al. [19]	None reported	1	Urination normal after six months; continuous vaginal dilation used to prevent stenosis.
7	Ulusoy et al. [20]	None reported	7	Electromyography and uroflowmetric tests showed normal function; pelvic EMG was also normal in all patients.
8	Ellerkamp, V et al. [21]	None reported	13	Uneventful outcomes post-surgery.
9	Yang et al. [22]	None reported	1	-
